# Nonlethal Furfural Exposure Causes Genomic Alterations and Adaptability Evolution in Saccharomyces cerevisiae

**DOI:** 10.1128/spectrum.01216-23

**Published:** 2023-07-03

**Authors:** Lei Qi, Ying-Xuan Zhu, Ye-Ke Wang, Xing-Xing Tang, Ke-Jing Li, Min He, Yang Sui, Pin-Mei Wang, Dao-Qiong Zheng, Ke Zhang

**Affiliations:** a Donghai Laboratory, Zhoushan, China; b Ocean College, Zhejiang University, Zhoushan, China; c Department of Molecular Genetics and Microbiology, Duke University, Durham, North Carolina, USA; d Life Sciences Institute, Zhejiang University, Hangzhou, China; e College of Life Science, Zhejiang University, Hangzhou, China; Broad Institute

**Keywords:** chromosome stability, furfural, genomic instability, yeasts

## Abstract

Furfural is a major inhibitor found in lignocellulosic hydrolysate, a promising feedstock for the biofermentation industry. In this study, we aimed to investigate the potential impact of this furan-derived chemical on yeast genome integrity and phenotypic evolution by using genetic screening systems and high-throughput analyses. Our results showed that the rates of aneuploidy, chromosomal rearrangements (including large deletions and duplications), and loss of heterozygosity (LOH) increased by 50-fold, 23-fold, and 4-fold, respectively, when yeast cells were cultured in medium containing a nonlethal dose of furfural (0.6 g/L). We observed significantly different ratios of genetic events between untreated and furfural-exposed cells, indicating that furfural exposure induced a unique pattern of genomic instability. Furfural exposure also increased the proportion of CG-to-TA and CG-to-AT base substitutions among point mutations, which was correlated with DNA oxidative damage. Interestingly, although monosomy of chromosomes often results in the slower growth of yeast under spontaneous conditions, we found that monosomic chromosome IX contributed to the enhanced furfural tolerance. Additionally, terminal LOH events on the right arm of chromosome IV, which led to homozygosity of the *SSD1* allele, were associated with furfural resistance. This study sheds light on the mechanisms underlying the influence of furfural on yeast genome integrity and adaptability evolution.

**IMPORTANCE** Industrial microorganisms are often exposed to multiple environmental stressors and inhibitors during their application. This study demonstrates that nonlethal concentrations of furfural in the culture medium can significantly induce genome instability in the yeast Saccharomyces cerevisiae. Notably, furfural-exposed yeast cells displayed frequent chromosome aberrations, indicating the potent teratogenicity of this inhibitor. We identified specific genomic alterations, including monosomic chromosome IX and loss of heterozygosity of the right arm of chromosome IV, that confer furfural tolerance to a diploid S. cerevisiae strain. These findings enhance our understanding of how microorganisms evolve and adapt to stressful environments and offer insights for developing strategies to improve their performance in industrial applications.

## INTRODUCTION

Furfural (2-furaldehyde) is a common dehydration by-product of xylose found in certain processed foods and beverages (1) and is the primary inhibitor present in cellulosic bioethanol production (2–4). Its concentrations in lignocellulosic hydrolysates typically range from 0.2 to 5 g/L, depending on the pretreatment techniques used (5). Furfural is also considered a hazardous contaminant in aquatic environments (6, 7). Understanding the genetic toxicity of furfural is crucial for comprehending its threats to the environment and for developing robust industrial microbial strains.

Chemicals that are genotoxic can cause alterations to an organism’s genome. These alterations may include point mutations, gene conversion, crossovers, chromosomal rearrangements (including large deletions and duplications), and aneuploidy. Previous studies have shown that furfural has mutagenic properties. For example, Zdzienicka et al. demonstrated the mutagenicity of furfural in Salmonella enterica serovar Typhimurium strain TA100 (8). Furfural has also been found to be positive in a forward-mutation assay for mouse lymphoma cells (9). Additionally, furfural was observed to cause chromatid breaks and exchanges in Chinese hamster ovary (CHO) cells and human lymphocytes that were treated with the compound (10, 11). Muñoz and Mazar Barnett found that Drosophila melanogaster females treated with furfural exhibited meiotic nondisjunction (12). Although these studies suggest that furfural exposure induces genome instability, the genetic mechanisms underlying these effects are still unknown. Also, little is known about how or whether furfural in biofermentation media impacts the genomic integrity of microorganisms.

Saccharomyces cerevisiae is a widely used model microorganism for assessing the genetic toxicity of drugs and environmental contaminants (13–18). Historically, research in this field had focused on measuring the rates of point mutations and DNA recombination using specific reporter genes (e.g., *URA3* and *CAN1*) that generate selectable phenotypes (19). However, with advancements in high-throughput methods such as DNA microarrays and next-generation sequencing, genomic alterations at the whole-genome level can now be detected (16, 20, 21). In a recent study, we found that the exposure of yeast cells to a lethal concentration (20 g/L) of furfural for 2 h led to the formation of about 20 DNA double-strand breaks (DSBs) per genome, which subsequently triggered frequent mitotic recombination throughout the yeast genome (13). It is worth noting that the concentrations of furfural typically found in the environment and lignocellulosic hydrolysates are much lower than 20 g/L (7, 13, 22). Whether or how nonlethal doses of furfural can affect yeast genomic integrity was unknown.

In this study, we investigated the global genomic alterations, ranging from point mutations to aneuploidy events, of S. cerevisiae isolates subcultured on plates containing nonlethal doses (<1 g/L) of furfural. We found that chronic exposure to low concentrations of furfural resulted in a distinct pattern of chromosomal instability compared to both untreated and 20-g/L furfural-treated cells. We also show that yeast isolates grown in furfural-containing medium were able to easily develop enhanced furfural tolerance. Finally, we identify certain genetic alterations that contribute to improved furfural tolerance.

## RESULTS

### Exposure to nonlethal concentrations of furfural results in chromosomal instability of S. cerevisiae.

**FIG 1:**
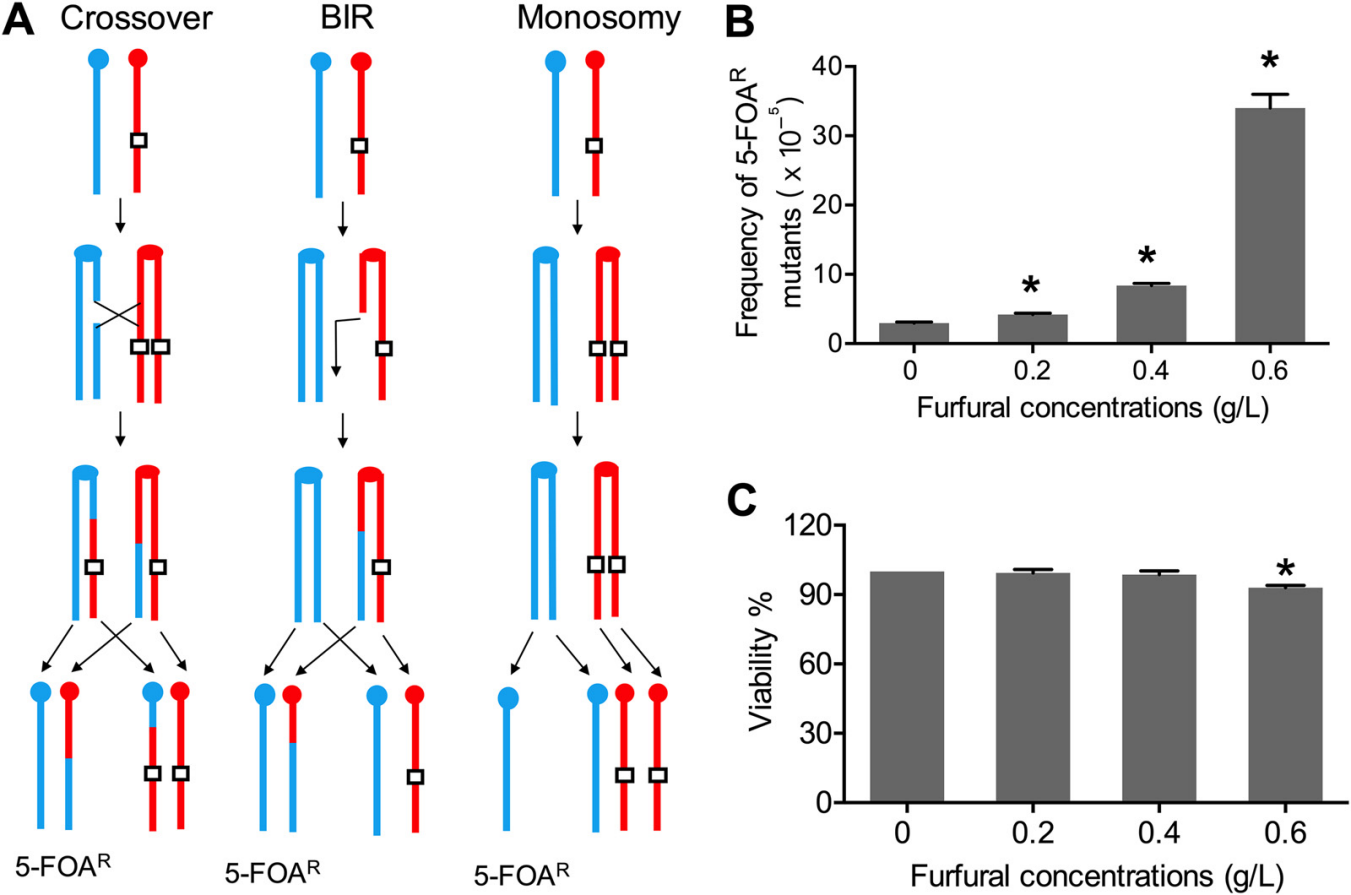
Detection of furfural-induced mitotic recombination. (A) Genetic mechanisms underlying the generation of 5-FOA-resistant (5-FOA^R^) colonies derived from the diploid strain WYUα. Red and blue lines represent W303-1A- and YJM789-derived homologs, respectively. Centromeres are depicted as ovals or circles, and in this strain, the *URA3* gene (rectangles) was inserted at the right arm of W303-1A-derived chromosome IV. Reciprocal crossover and break-induced replication (BIR), which are initiated by double-strand breaks, would lead to 5-FOA^R^ colonies. The loss of W303-1A-derived chromosome IV also results in a 5-FOA^R^ phenotype. PCR diagnosis can distinguish monosomy from crossover/BIR. (B) The frequency of 5-FOA^R^ colonies was measured when strain WYUα was grown on furfural-containing plates (for details, see Materials and Methods). (C) The cell viability of WYUα grown on plates containing furfural was also measured. Experiments in panels B and C were performed three times, and means ± standard deviations (SD) are shown. * indicates a significant difference (*P < *0.05) as evaluated by a *t* test.

To assess the effect of nonlethal doses of furfural on chromosomal stability, a genetic assay based on the selection of 5-fluoroorotic acid (5-FOA)-resistant (5-FOA^R^) mutants of S. cerevisiae strain WYUα was used. WYUα was constructed by mating haploid strains that were isogenic to W303-1A and YJM789 (see Table S1 in the supplemental material). We inserted a *URA3* gene at bp 1168918 on the right arm of W303-1A-derived chromosome IV. A reciprocal crossover or break-induced replication (BIR) event occurring between the centromere and *URA3* gave rise to a 5-FOA^R^ daughter cell (Fig. 1A). We cultured WYUα cells on nutrient-rich medium (yeast extract-peptone-dextrose [YPD] medium) without furfural and observed a frequency of 5-FOA^R^ mutants of 2.3 × 10^−5^ (Fig. 1B). However, the introduction of furfural at 0.2 g/L, 0.4 g/L, and 0.6 g/L increased the frequency of 5-FOA^R^ mutants by 2-, 4-, and 11-fold, respectively (Fig. 1B), with negligible effects on cell viability (Fig. 1C). It is important to note that the loss of W303-1A-derived chromosome IV in WYUα could also lead to 5-FOA^R^ colonies (Fig. 1A), which could be diagnosed by PCR (Table S2). We analyzed 60 5-FOA^R^ colonies isolated from YPD plates and found no instances of W303-1A-derived chromosome IV loss. However, 6 of 62 5-FOA^R^ mutants from plates containing 0.6 g/L furfural had lost the W303-1A-derived chromosome IV, indicating that 10% of 5-FOA^R^ mutants obtained on furfural-containing plates were the result of monosomy events. Our study shows that nonlethal doses of furfural can lead to mitotic recombination and chromosomal abnormalities in yeast.

### Genome-wide mapping of furfural-induced genetic events among subcultured isolates.

To detect furfural-induced genetic events at the whole-genome level, 31 isolates derived from the diploid strain JSC25-1 (23), which is isogenic to WYUα, were subcultured on YPD plates containing 0.6 g/L furfural. Three isolates were incubated on furfural plates for 1 generation, while the other 28 isolates were subcultured for 8 cycles to accumulate genomic alterations. The 31 isolates were then sequenced (21 isolates) or analyzed by a whole-genome single nucleotide polymorphism (SNP) microarray (10 isolates) (Data Set S1-1).

### (i) Furfural-induced loss of heterozygosity.

**FIG 2:**
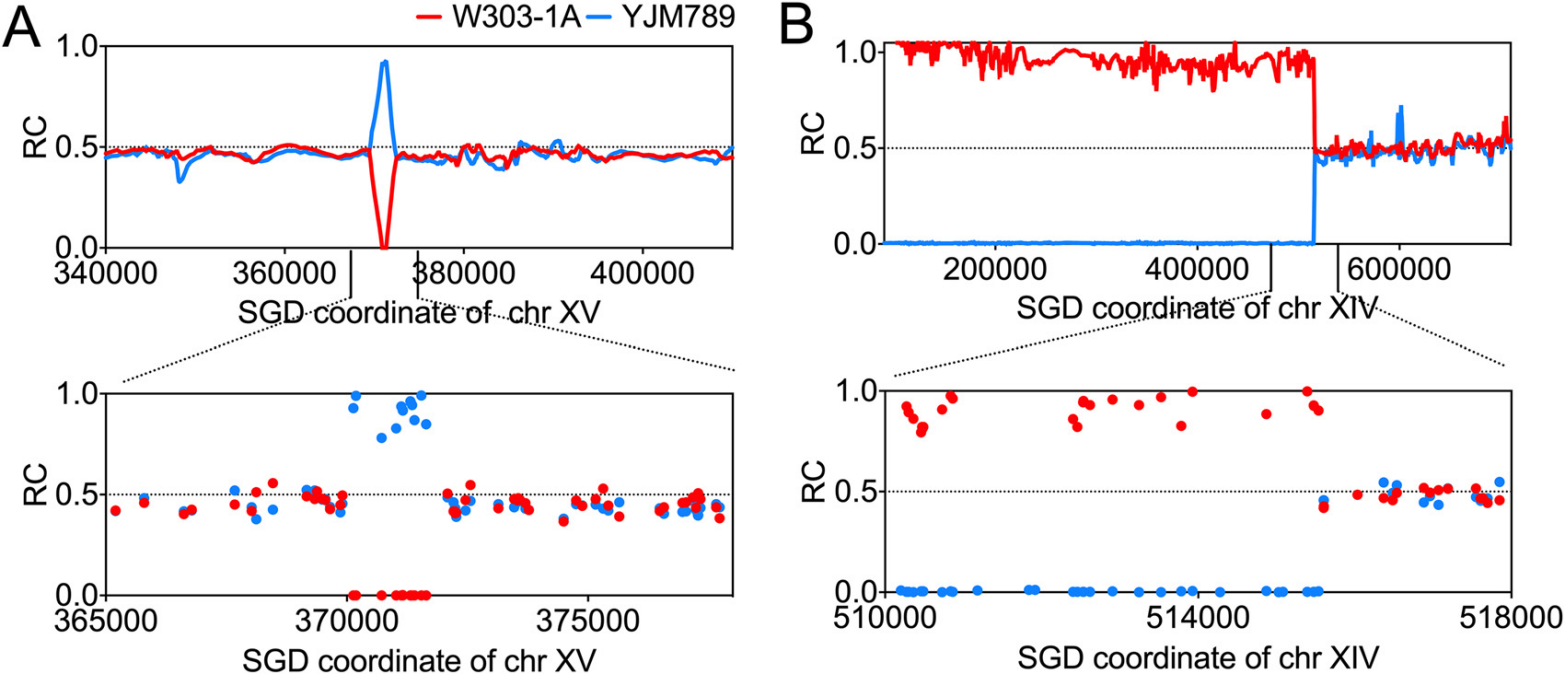
Examples of loss-of-heterozygosity (LOH) events detected in isolates subcultured on furfural-containing medium. The *y* axis values represent the relative coverage (RC), and the *x* axis values represent the coordinates of the chromosomes based on Saccharomyces Genome Database (SGD). RC values of 0, 0.5, and 1 indicate 0, 1, and 2 copies of W303-1A- and YJM789-derived homologs (red and blue lines, respectively). (A) Whole-genome sequencing of the LF8 isolate revealed an interstitial LOH (gene conversion) event on chromosome XV (chr XV). The breakpoint of this event is shown at high resolution below. (B) A terminal LOH event on chromosome XIV was observed in the LF8 isolate. The breakpoint of this event is also shown at high resolution.

Homologous recombination (HR) is the major mechanism for repairing DNA double-strand breaks (DSBs) in diploid yeast cells (24). Although HR makes it possible for cells to survive DSBs, when the homologous chromosome is used as the template to repair DSBs, it invariably leads to loss-of-heterozygosity (LOH) events (24). In the diploid strain JSC25-1, there are about 50,000 SNPs between two homologs across the whole genome; the abundance of SNPs enables the high-resolution identification of LOH events (23, 25). Figure 2 depicts two examples of LOH events, with red and blue dots indicating the relative coverage (RC) of W303-1A- and YJM789-specific SNPs, respectively. RC was calculated by dividing the coverage of each homolog-specific SNP by the average sequencing coverage for all SNPs in nonrepeat sequences. Consequently, RC values of 1, 0.5, and 0 indicate 2, 1, and 0 copies of homolog-specific sequences, respectively. In Fig. 2A, the copy number of YJM789-specific SNPs within the region from bp 369905 to bp 372085 on chromosome XV is 2, while the copy number of W303-1A-derived SNPs is 0. This pattern was identified as interstitial LOH (I-LOH), which could be explained by the repair of a DSB located between bp 369905 and bp 372085 on W303-1A-derived chromosome XV using the YJM789-derived homolog as the template (Fig. 2A). Figure 2B depicts a terminal LOH (T-LOH) event in which the homologous region was extended from the breakpoint to the left telomere of chromosome XIV. This T-LOH could have been caused by a reciprocal crossover or BIR (Fig. 1A).

**FIG 3:**
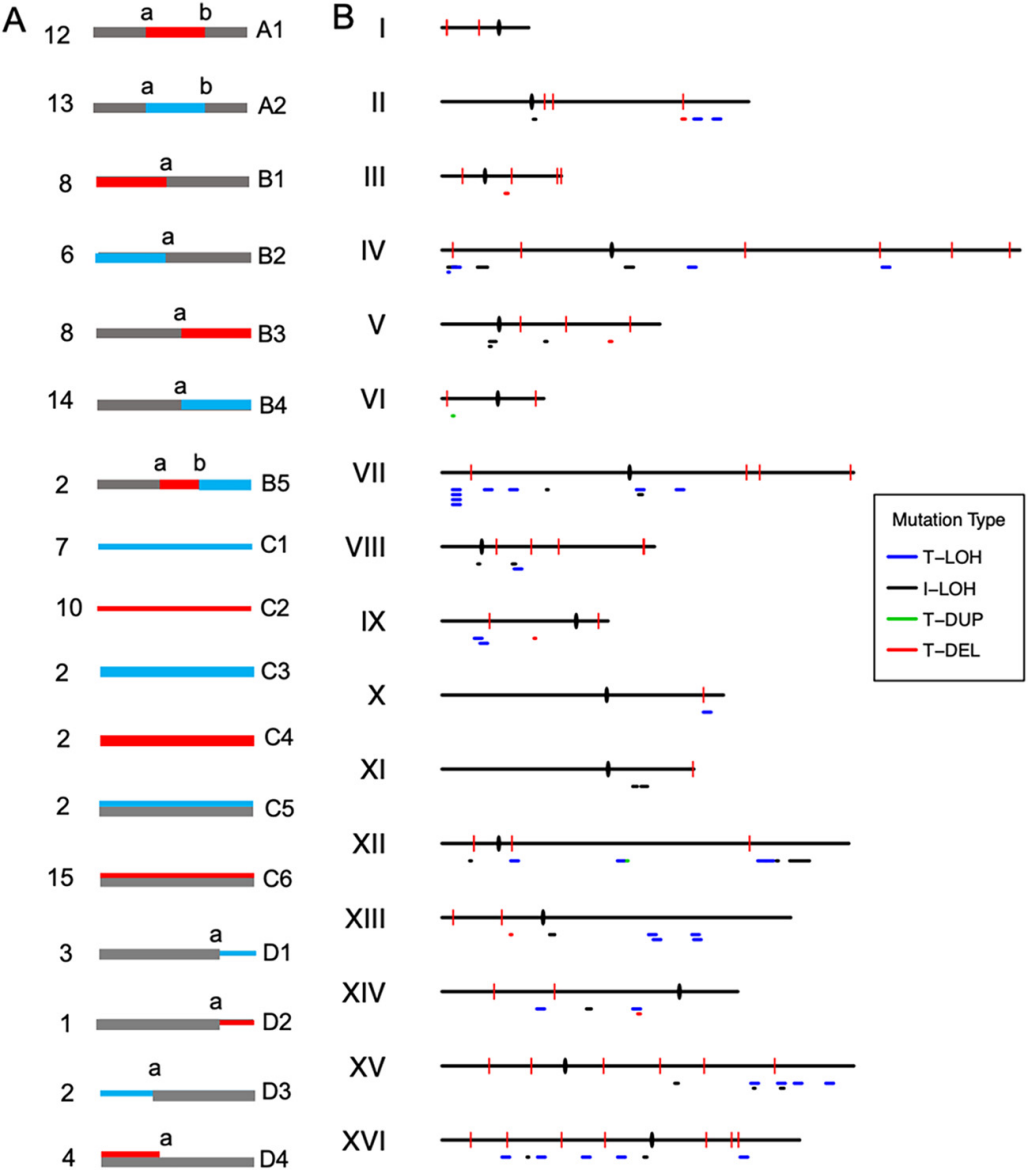
Patterns and distributions of genomic alterations induced by long-term exposure to nonlethal doses of furfural. (A) Various genomic alterations detected in subcultured isolates grouped and classified by type (classes A1 to D4). Red and blue lines indicate W303-1A- and YJM789-derived homologs, respectively, and gray lines indicate heterozygous regions. The numbers on the left indicate the number of events detected in subcultured strains for each class. The transitions between heterozygosity and homozygosity are labeled using lowercase letters. (B) Distribution of furfural-induced mitotic recombination events, including T-LOH, I-LOH, terminal duplication (T-DUP), and terminal deletion (T-DEL), across the 16 chromosomes. Single nucleotide variations and small insertions and deletions are shown as red vertical lines. Centromeres are shown as black ovals.

We identified 64 LOH events among the 31 subcultured isolates, including 25 I-LOH and 39 T-LOH events (Data Set S1). The patterns of all LOH events are shown in Fig. 3A. We further classified I-LOH as either class A1 or A2, depending on which homolog was used as the repairing template (Fig. 3A). Simple T-LOH events with a single transition between heterozygous and homozygous regions were classified as classes B1 to B4, while class B5 depicted gene conversion associated with T-LOH (Fig. 3A). Figure 3B shows the distribution of the 64 LOH events across the 16 chromosomes; no obvious recombination hot spots were observed. Based on the number of events, the number of cycles of cell division (~25 cell divisions during the formation of a colony from a single cell), and the number of analyzed isolates, it was calculated that the rates of I- and T-LOH were 2.2 × 10^−3^ and 3.4 × 10^−3^ events per genome per cell division, respectively. In a control experiment, 20 JSC25-1-derived isolates were subcultured on YPD plates for 20 cycles (500 cell divisions) before sequencing. Twelve I-LOH and four T-LOH events were detected (Data Set S1-2) in these 20 untreated isolates, indicating that the rates of spontaneous I-LOH and T-LOH events per genome per cell division are approximately 1.2 × 10^−3^ and 4 × 10^−4^, respectively. Our results show that the addition of 0.6 g/L furfural increased the rate of T-LOH in JSC25-1 by 8.5-fold but increased the rate of I-LOH by only 1.8-fold. Using Fisher’s exact test, we found that the ratio of T-LOH to I-LOH in furfural-treated isolates is significantly higher than that in untreated isolates (*P < *0.05). A possible explanation for the altered ratio of T- to I-LOH is discussed in Discussion.

### (ii) Large-scale chromosomal rearrangements.

**FIG 4:**
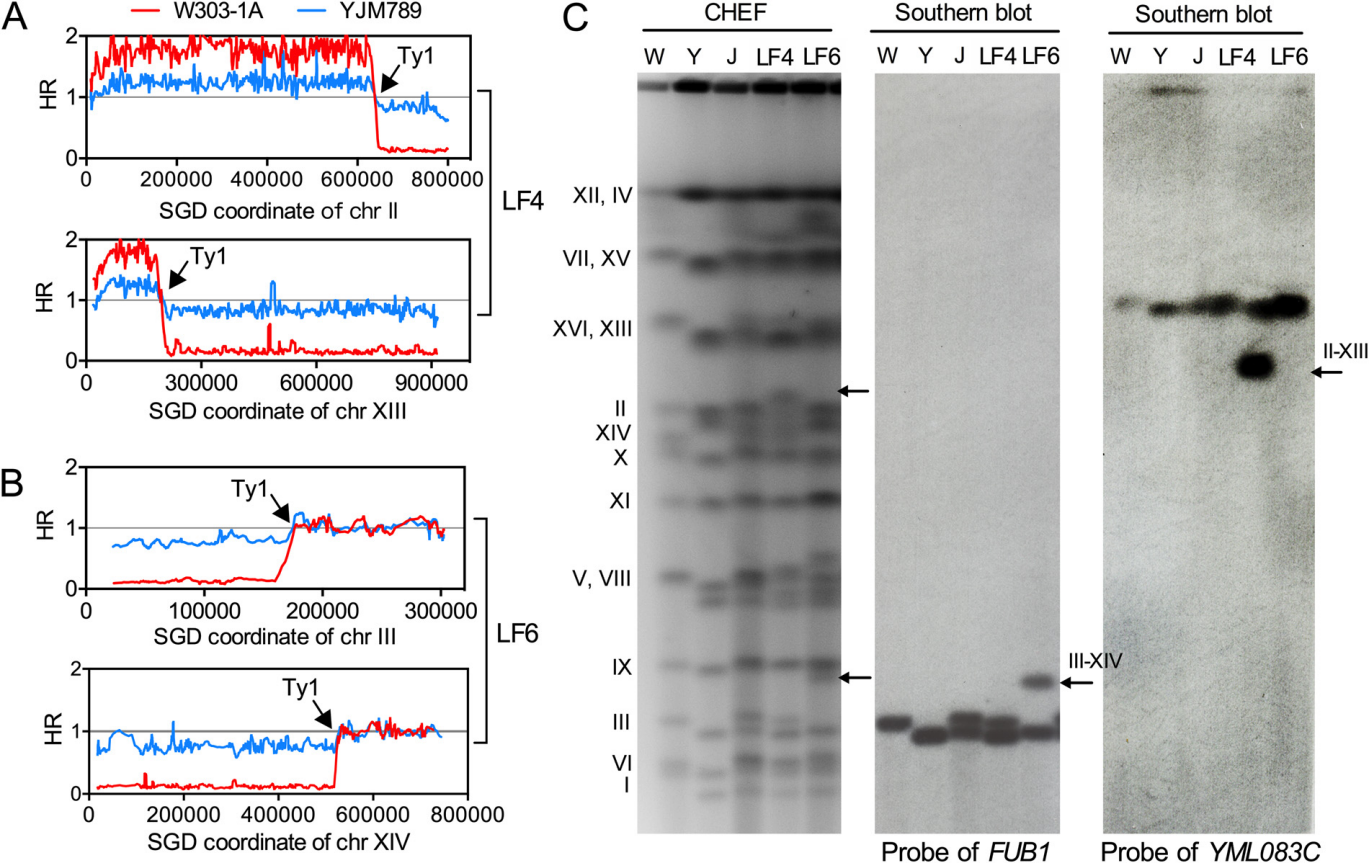
Furfural-induced translocations among subcultured isolates. (A) The *y* axis represents the hybridization ratio, and the *x* axis represents the coordinates of the chromosomes, with HR values of 0.2, 1, and 1.5 indicating 0, 1, and 2 copies of W303-1A- and YJM789-derived homologs (red and blue lines, respectively). Microarray results for chromosomes II and XIII in isolate LF4 showed partial duplications of W303-1A-derived chromosome II (kb 0 to 644) and chromosome XIII (kb 0 to 183). (B) Two deletion events were observed on W303-1A-derived chromosome III (kb 0 to 175) and chromosome XIV (kb 0 to 519) in isolate LF6. (C) Pulsed-field gel electrophoresis (PFGE) and Southern blot analysis of isolates LF4 and LF6 using strains W303-1A, YJM789, and JSC25-1 (indicated by W, Y, and J, respectively). Yeast chromosomes were separated by PFGE. The novel band with a size of 818 kb (indicated by black arrows) resulted from a translocation event between chromosomes II and XIII. The 818-kb band also probes to gene *YML083C* (at kb 100 on chromosome XIII). The recombined chromosome III-XIV (~400 kb) was confirmed by using a probe that targets gene *FUB1* (at kb 250 on chromosome III).

In contrast to copy-number-neutral LOH events, chromosomal rearrangements typically result in substantial deletions and duplications. In these cases, the copy number of one homolog remains unchanged, whereas the other homolog is either deleted or duplicated. As shown in Fig. 4A, the SNP microarray pattern showed that two chromosomal regions (bp 1 to 635650 of chromosome II and bp 1 to 182683 of chromosome XIII) were amplified, and two regions (bp 644285 to the right end of chromosome II and bp 183803 to the right end of chromosome XIII) were deleted in isolate LF4. According to our previous work (26), the majority of “paired” terminal deletions and duplications are the results of translocations between a centromere-containing fragment and an acentric fragment to form monocentric recombined chromosomes. In the LF4 lane of the pulsed-field gel electrophoresis (PFGE) results, a new band with a size of 818 kb was detected (Fig. 4C). It is likely that the segment (bp 1 to 182683) of chromosome XIII was translocated to the centromere-containing segment (bp 1 to 635650) of chromosome II, resulting in a novel chromosome with an observed size of 818 kb. Southern blotting with a probe targeting the *YML083C* gene verified this translocation event (Fig. 4C). In isolate LF6, there were 2 terminal deletions on chromosome III (bp 1 to 175342) and chromosome XIV (bp 1 to 518591) but no terminal duplications (Fig. 4B). We anticipated that the remaining segments of chromosomes III and XIV recombined to form a stable, 400-kb monocentric chromosome. The novel chromosome was validated by Southern blotting with a probe specific for the *FUB1* gene (Fig. 4C). The presence of Ty elements at the breakpoints of these rearrangements suggests that repeat-mediated HR was responsible for these translocations. Similarly, two terminal deletion events in isolate LF20 could also be explained by recombination between ectopic repeats on YJM789-derived chromosome V and W303-1A-derived chromosome IX (Fig. S1). In Fig. S2, we present a potential mechanism for the paired deletions depicted in Fig. 4B. Initially, a DSB close to a repeat site (shown by black arrows) triggered HR by utilizing a nonallelic homolog as the template. Next, two nonhomologous chromosomes engaged in a reciprocal crossover created translocation events. Finally, the nondisjunction of the recombined chromatids (indicated by chromatids 3 and 4) resulted in two deletions in one daughter cell.

In summary, we identified 8 large (>50-kb) chromosomal rearrangements, including 6 terminal deletions and 2 terminal duplications, in the 31 isolates subcultured on furfural plates (Fig. 3A and Data Set S1). The rate of large-scale rearrangements was estimated to be 7 × 10^−4^ events per genome per cell division, which is about 23-fold higher than that of the spontaneous situation (20). Our analysis suggests that exposure to a nonlethal concentration of furfural increased the rate of chromosomal rearrangement by a factor of at least 10.

### (iii) Whole-chromosome aneuploidy in subcultured isolates.

Apart from LOH and chromosomal rearrangements, we also analyzed the presence of aneuploidy events in the 31 isolates, among these 31 strains, we identified 15 trisomic and 17 monosomic chromosomes, as well as 4 unique parental disomy (UPD) events (Fig. 3A and Data Set S1-3). In UPD events, one homolog is lost, and the other is duplicated (27). The rate of chromosomal aneuploidy in isolates grown on furfural (0.6 g/L) plates was 3.2 × 10^−3^ events per genome per cell division, which was about 50-fold higher than that of a wild-type strain under spontaneous conditions (20). In contrast to our recent study, which found trisomic chromosomes to be the most prevalent aneuploidy events in wild-type cells subcultured on YPD plates (20), most furfural-treated isolates exhibited monosomic chromosomes, except for isolate LF18, which had 7 trisomic chromosomes (Data Set S1-3). Of the 17 monosomy events, 7 were monosomic chromosome IX, 4 were monosomic chromosome V, and 4 were monosomic chromosome III (Data Set S1-3). Our findings suggest that certain chromosomes are more susceptible to loss in furfural-treated cells, leading to aneuploidy.

**FIG 5:**
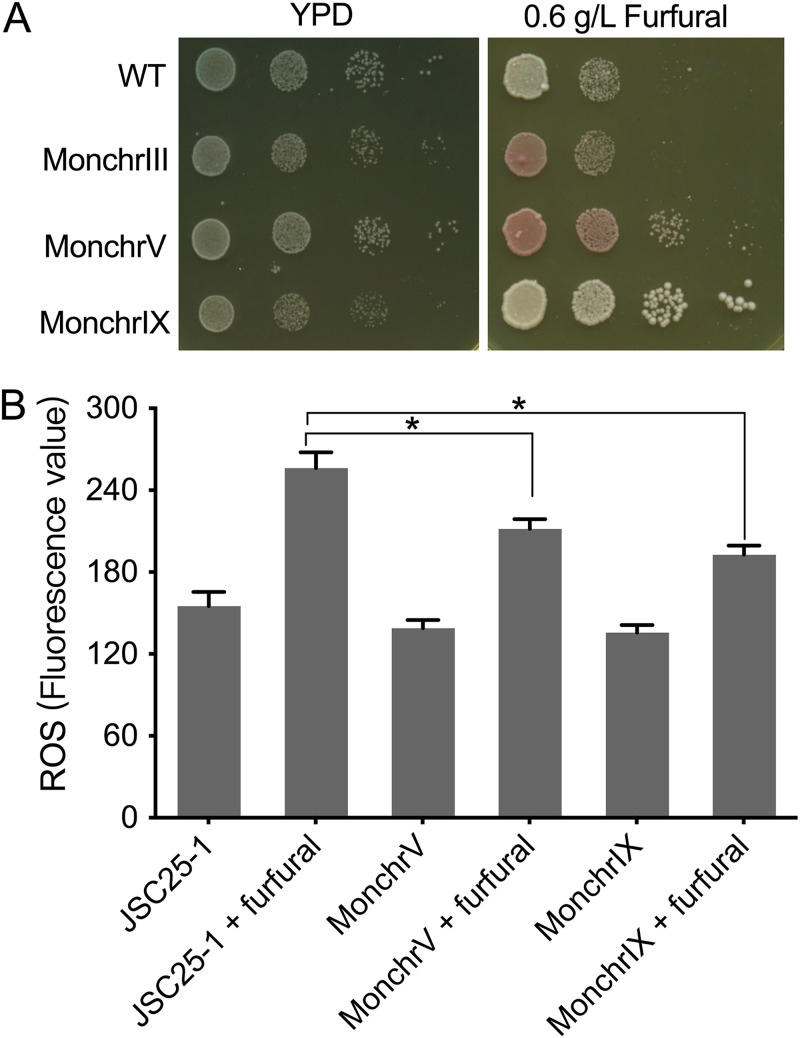
Aneuploidy events alter furfural tolerance in yeast. The control wild-type (WT) strain is JSC25-1, and MonchrIII, MonchrV, and MonchrIX are JSC25-1-isogenic mutants with monosomic chromosomes III, V, and IX, respectively. (A) The aneuploid MonchrIX mutant showed improved furfural tolerance compared to JSC25-1. (B) Furfural-induced ROS accumulation was measured in cells of the euploid strain JSC25-1 and the aneuploid MonchrIX mutant that were precultured in YPD medium and then transferred to 1× PBS buffer with and without 0.6 g/L furfural. After 1 h of treatment, ROS were detected by a fluorescence spectrophotometer. (C) NADH-dependent furfural reductase activity was measured in yeast cells precultured in YPD medium and then transferred to fresh YPD medium and YPD medium with 0.6 g/L furfural. Total proteins were extracted at the 0- and 2-h time points, and furfural reduction activity was determined by measuring the decreased absorbance at 340 nm using a microplate reader. * indicates a significant difference at a significance level of a *P* value of 0.05 as evaluated by the *t* test.

To investigate the effects of monosomy events on furfural tolerance, we created three isogenic aneuploidy mutants (MonchrIII, MonchrV, and MonchrIX) of the JSC25-1 strain, each with monosomic chromosomes III, V, and IX, respectively. In the absence of furfural, MonchrV had a growth rate similar to that of its diploid parent strain, while MonchrIII and MonchrIX grew more slowly than their parent (Fig. 5A). However, when grown on plates containing 0.6 g/L of furfural, MonchrIX outperformed the diploid strain (Fig. 5A). We observed that MonchrIII and MonchrV exhibited red colonies when exposed to 0.6 g/L furfural. These two mutants were homozygous for the *ade2-1* allele, which allows the accumulation of red pigment (a precursor of adenine) in yeast cells. Under normal conditions on YPD plates, MonchrIII and MonchrV displayed pink colonies due to the presence of a copy of the *SUP4*-o gene on chromosome IV, which partially suppressed the effect of the *ade2-1* mutation. It is likely that furfural treatment resulted in the increased activity of the adenine synthesis pathway and the accumulation of purine precursors through an unclear physiological mechanism. Overall, the growth advantage of MonchrIX on furfural plates suggests that certain monosomy events may confer enhanced furfural tolerance to a diploid S. cerevisiae strain.

Reduced cellular reducing power and the accumulation of reactive oxygen species (ROS) have been identified as primary physiological factors affecting yeast cell growth and viability under furfural stress (2, 28). By staining yeast cells with 2′,7′-dichlorodihydrofluorescein diacetate (DCFH-DA), we found that cells treated with 0.6 g/L of furfural accumulated 44% more ROS than untreated cells (Fig. 5B). Furfural exposure stimulated ROS accumulation in both wild-type and MonchrIX cells, but the ROS level in MonchrIX was 20% lower than that in the wild-type strain. The conversion of furfural to the less toxic 2-furanmethanol by oxidoreductases, using the NADH cofactor, is essential for furfural resistance in yeast (29). MonchrIX showed 3.7- and 2.3-fold-higher furfural reduction activities than the diploid strain in YPD medium and YPD medium containing 0.6 g/L furfural, respectively (Fig. 5C). These findings show that yeast cells that lost one copy of chromosome IX had a stronger capacity to detoxify furfural, resulting in decreased ROS accumulation and enhanced growth under furfural stress.

### (iv) Furfural-induced point mutations.

We identified 56 point mutations among the 21 sequenced isolates subcultured on furfural plates, consisting of 53 single nucleotide variations (SNVs) and 3 small insertions/deletions (indels) (Fig. 3B and Table S3). By calculating mutation rates based on genome size (24 Mb) and the number of cell divisions before sequencing, we estimated that the rates of SNVs and indels were 3 × 10^−10^ and 1.7 × 10^−11^ per base per cell division, respectively, among the furfural-treated isolates. In comparison, the rates of SNVs and indels were about 2.1 × 10^−10^ and 2.2 × 10^−11^ per base per cell division, respectively, when wild-type S. cerevisiae cells were incubated on YPD plates (20). Therefore, it is likely that exposure to 0.6 g/L of furfural induced a modest increase in the SNV rate. Out of the 53 SNVs, 4 were found to be located within the LOH tract of a total size of 557,567 bp for these sequenced isolates. Assuming a random distribution of SNVs across the genome, we would have expected 1.2 SNVs within the LOH region (calculated as 557,567/24,000,000 × 53 = 1.2). The chi-square test revealed a significant enrichment of SNVs within the LOH region in the furfural-treated isolates (*P < *0.01).

**FIG 6:**
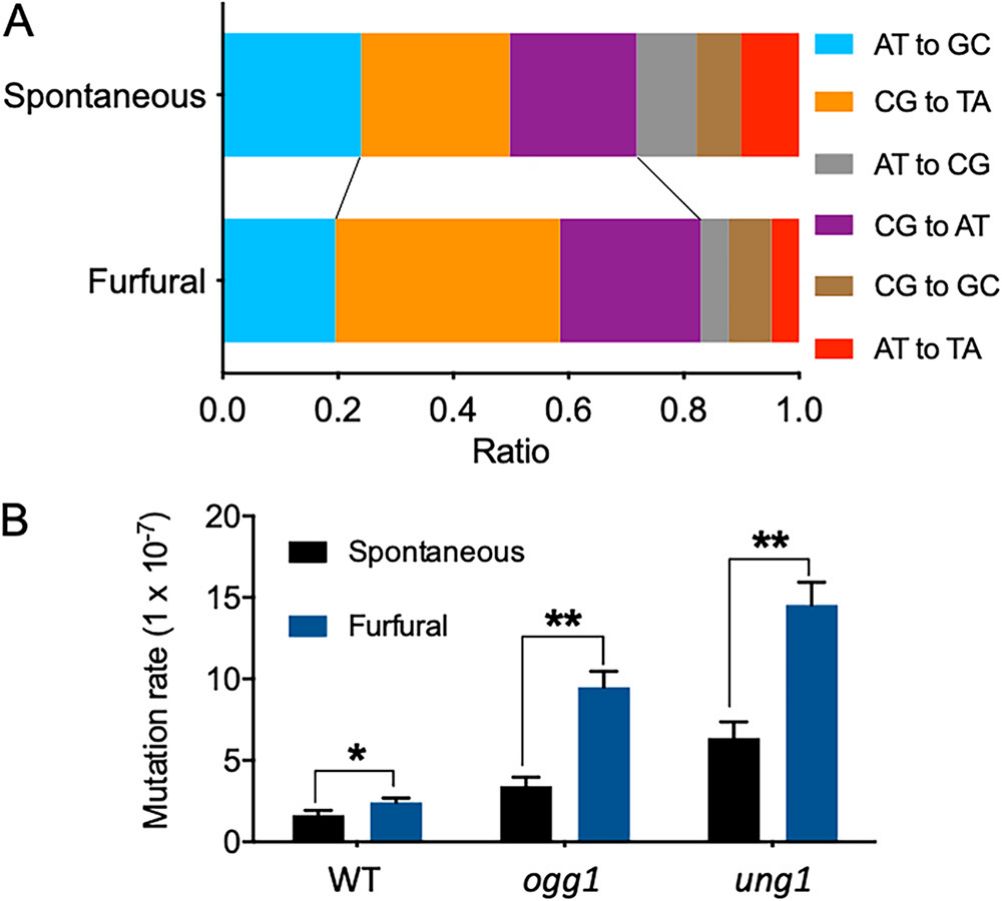
Furfural-induced base substitutions. (A) Spectrum of SNVs detected in untreated (20) and furfural-treated JSC25-1-derived isolates. The cumulative ratio of CG-to-TA and CG-to-AT base substitutions was up to 60% in furfural-treated isolates (*P < *0.05 by Fisher’s exact test). (B) Comparison of rates of mutation of the *CAN1* gene in the wild type and glycosylase-deficient mutants (*Ogg1* and *Ung1*) under spontaneous and furfural-exposed conditions. All values were compared to the values for the wild-type strain by a *t* test, and significant differences are shown by single asterisks (*P < *0.05) or double asterisks (*P < *0.01).

Further analysis was performed on the six potential types of mutations, and CG-to-AT and CG-to-TA mutations were responsible for 26% and 22% of SNVs, respectively, in untreated cells (Fig. 6A). The cumulative ratio of these two types of base substitution increased to 60% in furfural-treated isolates (*P < *0.05 by Fisher’s exact test) (Fig. 6A). Previous studies have linked CG-to-AT and CG-to-TA mutations to oxidative damage to C and G bases (30, 31). The base excision repair (BER) pathway has been identified as the principal mechanism for repairing oxidized bases, and it is typically initiated by DNA glycosylases (32). The glycosylase Ogg1 excises 7,8-dihydro-8-oxoguanine (8-oxo-G) residues located opposite cytosine or thymine residues in DNA, whereas Ung1 excises uracil from single-stranded DNA (ssDNA) (32). In this study, we measured the rates of mutation of the *CAN1* gene in a wild-type haploid strain, MC42-2d, and the DNA glycosylase-deficient mutants SY112 (*ogg1*-null mutant) and SY113 (*ung1*-null mutant) to determine whether BER was involved in mutagenesis caused by furfural. The frequency of the *can1* mutation in MC42-2d was 1.5-fold higher on furfural plates than on YPD plates (Fig. 6B). The *ogg1* and *ung1* mutants displayed 2- and 4-fold-higher mutation frequencies than the wild-type strain on YPD plates (Fig. 6B), respectively, indicating that both Ogg1 and Ung1 play a role in preventing spontaneous mutations in wild-type cells. In the presence of furfural, the *ogg1* and *ung1* mutants had 5- and 7-fold-higher frequencies of the *can1* mutation than in its absence, respectively (*P < *0.001 by a *t* test) (Fig. 6B). The increased mutagenic effect of furfural on the *ogg1* and *ung1* mutants indicated that furfural treatment increased 8-oxo-G and uracil incorporation into DNA. These results demonstrate that the BER pathway is crucial for preventing mutations induced by furfural exposure.

### T-LOH events on chromosome IV altered furfural tolerance.

**FIG 7:**
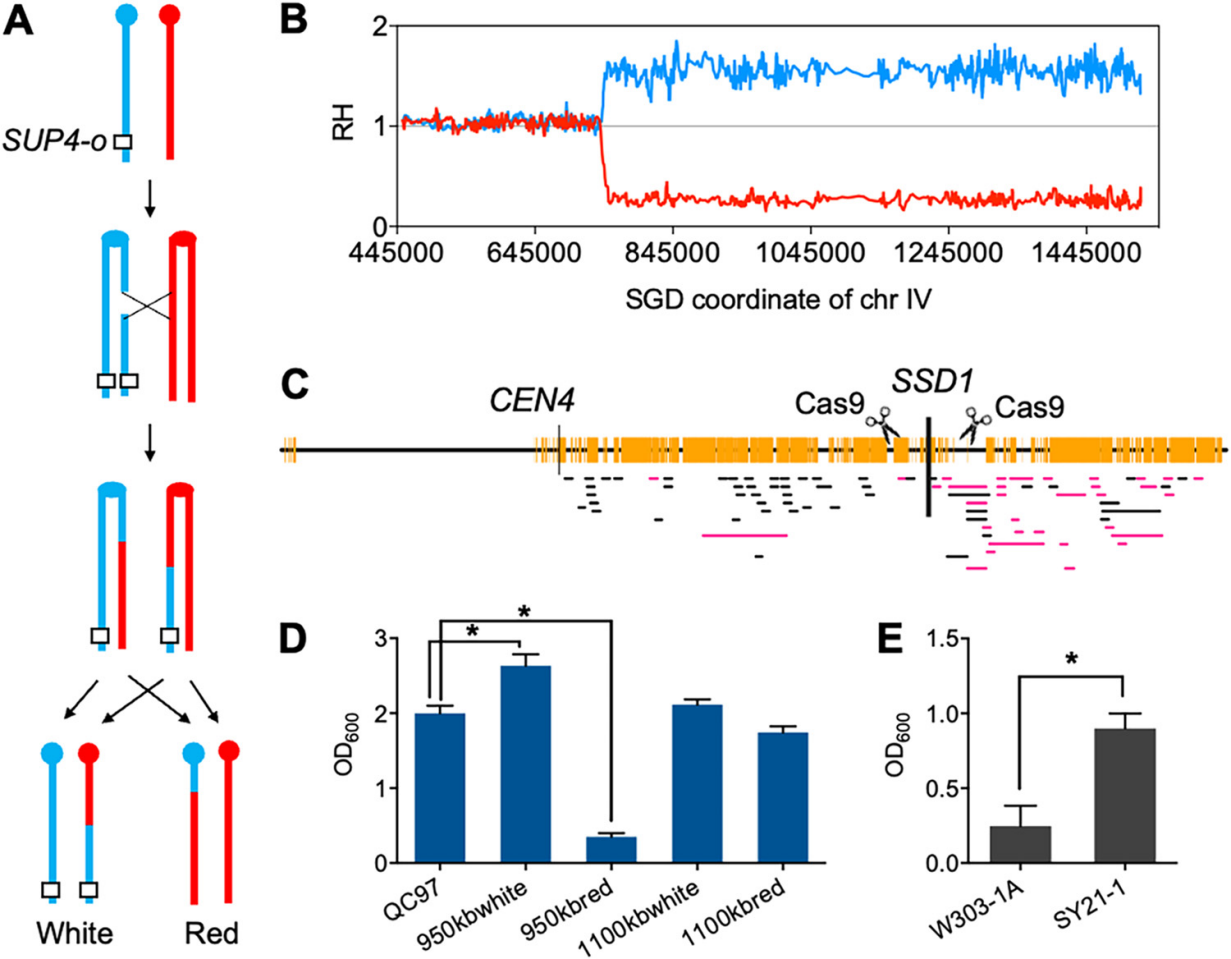
Analysis of furfural-induced T-LOH events on the right arm of chromosome IV. Red and blue lines represent W303-1A- and YJM78-derived chromosomes, respectively. (A) T-LOH events were selected using a sectored colony screening system. In JSC25-1, the ochre-suppressing gene *SUP4*-o was inserted on the right end of YJM789-derived chromosome IV. Reciprocal crossover results in white/red sectored colonies, while a BIR event leads to pink/red or pink/white colonies. (B) Analysis of a T-LOH event by a custom chromosome IV-specific SNP microarray. This array allows the identification of the breakpoint of a T-LOH event in the selected white colony. (C) Distributions of the breakpoints of furfural-induced T-LOH events on the right arm of chromosome IV. The yellow vertical lines indicate the SNP sites on this chromosome. The black and pink horizontal lines indicate the breakpoints of T-LOH events in the white and red colonies, respectively. The CRISPR/Cas9 system was used to construct T-LOH events with specific breakpoints through the introduction of DSBs at kb 950 and kb 1100 on the right arm of chromosome IV. (D) Comparison of furfural tolerance in QC97 and QC97-derived isolates. QC97 is isogenic to JSC25-1. Isolates 950kbwhite and 950kbred are homozygous for YJM7890- and W303-1A-derived sequences from about kb 950 to the right end of chromosome IV. Isolates 1100kbwhite and 1100kbred are homozygous for YJM7890- and W303-1A-derived sequences from about kb 1100 to the right end of chromosome IV. * indicates statistically different biomass formation in medium containing 0.6 g/L furfural compared to the QC97 control group. (E) In the presence of 0.6 g/L furfural, SY21-1 (W303-1A background haploid with a functional *SSD1* allele) showed improved furfural tolerance compared to W303-1A (with a nonfunctional *ssd1* allele). OD_600_, optical density at 600 nm.

JSC25-1 carries the ochre allele *ade2-1* homozygously and a copy of *SUP4*-o on the right arm of YJM789-derived chromosome IV. This strain forms pink colonies on solid media. When crossovers occur between the centromere and *SUP4*-o in the first cell cycle after plating, sectored colonies can arise, with half white (two copies of *SUP4*-o) and half red (no *SUP4*-o) sectors (Fig. 7A). For untreated JSC25-1 cells, the frequency of white/red sectored colonies was about 4 × 10^−5^, and this frequency doubled in the presence of 0.6 g/L of furfural. However, we observed more fully white colonies (appearing at a frequency of about 7 × 10^−4^) than sectored colonies. We hypothesize that specific sequences’ homozygosity resulting from T-LOH events may lead the two daughter cells to have vastly different growth rates under furfural stress.

To verify this hypothesis, we mapped the breakpoints of T-LOH events in 53 independent white colonies and 33 independent red colonies using a custom chromosome IV-specific SNP microarray (23). This array is represented by 100 and 2,300 SNPs on the left and right arms of chromosome IV, respectively. If a crossover event occurred on the right arm of chromosome IV, the daughter cells’ sequences between the breakpoint and the telomere should be homozygous for either W303-1A or YJM789 (Fig. 7A). An example of a T-LOH event is shown in Fig. 7B, where a signal transition from heterozygosity to homozygosity of YJM789 sequences was found at approximately kb 745. Most T-LOH events in white colonies (74%) were found to have been initiated by DNA lesions on the left side (kb 450 to kb 1050) of the right arm of chromosome IV, whereas nearly all red colonies had breakpoints located between kb 1050 and the right end of chromosome IV (Fig. 7C). The coordinates of the T-LOH breakpoints are listed in Data Set S1-4. The biased distribution of the T-LOH breakpoints supports our hypothesis that the homozygosity of certain sequences on chromosome IV impacts yeast furfural tolerance.

To determine which region’s LOH accounted for the altered furfural tolerance, we generated four mutants by incising the right arm of chromosome IV using CRISPR/Cas9. Two guide RNA (gRNA) sequences were specifically designed to target kb 950 and kb 1100 on chromosome IV (Fig. 7C). The gRNA was expressed in a JSC25-1-isogenic strain, QC97, in which the *CAS9* gene, regulated by the *GAL1* promoter, was inserted at the *HIS3* locus on W303-1A-derived chromosome XV. After a 2-h incubation in 2% galactose medium, QC97 was plated onto YPD plates to screen for white/red sectored colonies. The frequency of sectored colonies caused by Cas9 incisions at kb 950 or kb 1100 was about 2%. The cells isolated from the white/red portions of the sectored colonies were termed 950kbwhite, 950kbred, 1100kbwhite, and 1100kbred based on the break sites and colony colors. Our results showed that 950white grew the fastest, followed by 1100kbwhite and 1100kbred, and that 950kbred grew the slowest in medium containing 0.6 g/L furfural (Fig. 7D). This finding demonstrates that different T-LOH events on the right arm of chromosome IV can contribute to variations in the furfural tolerance of yeast.

By analyzing the DNA sequences of genes located at approximately kb 1000 on chromosome IV in the W303-1A and YJM789 strains of yeast, we discovered that the *SSD1* gene at kb 1046 on chromosome IV was inactivated in the W303-1A background strain due to a premature stop codon. *SSD1* is a conserved fungal RNA-binding protein that has been genetically linked to various cellular processes, including cell wall integrity, protein homeostasis, and mitochondrial function maintenance (33). To determine the role of *SSD1* in furfural tolerance, we restored the nonfunctional *SSD1* allele on the W303-1A genome to a functional allele derived from YJM789 (see Materials and Methods). This manipulation significantly increased the furfural resistance of W303-1A, as shown in Fig. 7E. This result confirms that the normal function of Ssd1p is necessary for furfural tolerance in yeast, which explains why recombinants with a homologous W303-1A-derived *SSD1* allele were scarcely selected on furfural-containing plates. Thus, our results demonstrate that specific LOH events that impact furfural resistance can be strongly selected in diploid yeast when exposed to furfural-containing medium.

## DISCUSSION

This study utilized multiple genetic screening assays and high-throughput analysis approaches to investigate the genotoxic and evolutionary effects of nonlethal doses of furfural on yeast. Our primary findings are as follows: (i) exposure to furfural significantly increases the occurrence of chromosomal aneuploidy and mitotic recombination in yeast; (ii) certain events resulting in monosomy contribute to furfural resistance in yeast; (iii) T-LOH events are more prevalent than I-LOH events in furfural-treated cells, in contrast to untreated cells; (iv) furfural treatment leads to higher ratios of CG-to-AT and CG-to-TA substitutions, which are associated with base oxidation; and (v) certain LOH events on chromosome IV alter furfural tolerance in diploid yeast. Below, we discuss the implications of these findings.

### Chronic exposure to furfural has a potent inductive effect on chromosomal aneuploidy.

Genomic alterations serve as the molecular basis of phenotypic diversity in yeast populations. In a recent study, we determined the frequencies of various spontaneous genomic alterations in a wild-type diploid S. cerevisiae strain by genome sequencing of 93 yeast isolates subcultured on YPD medium for hundreds of generations (Table 1) (20). Compared to spontaneous conditions, the treatment of G_1_-phase synchronized yeast cells with a lethal concentration (20 g/L) of furfural resulted in significantly higher rates of genomic alterations; however, the relative rates of genetic events were not altered (Table 1) (13). In this study, we identified 103 genomic alterations among 31 JSC25-1-derived isolates grown on furfural (0.6 g/L)-containing plates. The rates of aneuploidy, chromosomal rearrangements, and LOH in these isolates were 50-, 23-, and 4-fold higher, respectively, than those in untreated cells (Table 1). Our findings are consistent with the teratogenic and recombinogenic effects observed in fly and human cell cultures (11, 34). Remarkably, 0.6 g/L furfural had a stronger induction effect on aneuploidy than LOH (Table 1).

**TABLE 1:** Relative ratios of different classes of genomic alterations

Condition	Total number of genomic alterations (ratio %)				
	Trisomic chromosomes	Monosomic chromosomes	Large deletions/duplications	I-LOH	T-LOH
Spontaneous^*a*^	12 (0.9)	3 (0.2)	47 (3.7)	859 (67.3)	356 (27.9)
20 g/L furfural^*b*^	3 (1.9)	0 (0.0)	4 (2.5)	109 (69.4)	41 (26.1)
0.6 g/L furfural	14 (1.4)	13 (13.1)	8 (8.1)	25 (25.3)	39 (39.4)

a A total of 93 WYspo11-derived isolates that were subcultured on YPD plates for 60 or 120 generations were sequenced.

b JSC25-1 cells collected from YPD plates were treated with 20 g/L furfural for 2 h and then plated onto YPD plates; such processes were repeated 18 times, and 21 independent isolates were analyzed.

Why did aneuploidy events occur frequently in yeast cells exposed to 0.6 g/L furfural? In eukaryotic cells, the replicated genome is packaged into mitotic chromosomes, and each contains two identical sister chromatids after entering mitosis. All chromosomes are then oriented to enable the segregation of sister chromatids toward the mitotic poles. Many factors, including errors in the control of sister chromatid cohesion, spindle assembly defects, and incomplete chromosome replication, can impede the partitioning of the two genomes and lead to chromosome segregation defects (35). Since furfural can act as a thiol-reactive electrophile and can react with the sulfhydryl groups present in proteins (36), it is possible that furfural can oxidize and interfere with the functions of proteins involved in chromosome segregation. Additionally, ROS are pleiotropic factors that can significantly increase chromosomal nondisjunction due to oxidative damage to the cohesion complex, impairing spindle formation and inhibiting kinetochore assembly (37). Therefore, furfural treatment-induced decreases in intracellular reductive power and ROS accumulation (13, 28) (Fig. 5B) could also exacerbate the occurrence of chromosomal nondisjunction.

### Improved furfural tolerance in the aneuploid strain with monosomic chromosome IX.

Due to the harmful nature of chromosomal loss in yeast cells, trisomy events are more common than monosomy events in untreated cells (20, 38). Previous studies have shown that the duplication of chromosomes III, V, and XI enhanced S. cerevisiae’s resistance to heat (39), high pH (39), and oxidative stress (40), demonstrating an unequivocal role for chromosome duplication in rapid stress adaptation. However, our research found that yeast cells subcultured on plates containing 0.6 g/L furfural were more inclined to lose chromosomes instead of gaining them. One plausible justification for this result is that certain monosomy events contribute to the fitness of yeast cells under furfural stress and thus were enriched in the subcultured isolates. Indeed, the aneuploid mutant with monosomic chromosome IX grew significantly faster than the parental diploid strain on furfural medium (Fig. 5A).

How did the loss of one copy of chromosome IX help yeast cells tolerate furfural? In S. cerevisiae, the toxic aldehyde group of furfural can be reduced to a hydroxyl group by multiple enzymes, including alcohol dehydrogenases (Adh1p, Adh6p, and Adh7p), methylglyoxal reductase (Ari1p), and aldehyde reductase (Gre3p), using NADH as the cofactor (36). Previous studies have linked the higher activity levels of these enzymes to enhanced furfural tolerance in multiple yeast strains (41–43). Our analysis suggests that the increased NADH-dependent furfural reduction ability and decreased ROS accumulation (Fig. 5) could, at least partially, contribute to the improved furfural tolerance in the monosomic chromosome IX mutants. However, the detailed physiological mechanism underlying the enhanced NADH-dependent furfural reduction activity remains to be investigated further.

### Possible mechanisms underlying the skewed ratio of T-LOH to I-LOH in yeast isolates subcultured on furfural medium.

Table 1 shows that exposure to 0.6 g/L furfural resulted in a higher T-LOH-to-I-LOH ratio in diploid yeast cells than under spontaneous and 20-g/L furfural treatment conditions. According to the established theory of mitotic recombination (44), I-LOH mainly reflects the repairing of DSBs through homologous recombination using the synthesis-dependent strand annealing (SDSA) pathway, while T-LOH could result from the double-strand break repair (DSBR) pathway or the BIR pathway. In wild-type cells, most spontaneous LOH events were initiated by DSBs occurring in the G_1_ phase (23). Our previous study showed that most G_1_-phase DSBs were repaired by the SDSA pathway and led to a majority of I-LOH in daughter cells (25). This observation explains why I-LOH is more frequently observed than T-LOH under spontaneous conditions (Table 1). In yeast cells treated with 20 g/L furfural for 2 h, ROS-mediated DSBs are the primary recombinational lesions (13). Since most of the treated cells were presynchronized at the G_1_ phase (13), it is not surprising that the ratio of I-LOH to T-LOH in 20-g/L furfural-treated cells was similar to that in untreated cells (Table 1).

In this study, the addition of 0.6 g/L furfural to media allowed yeast cells to divide, indicating that furfural may pose a threat to genome integrity throughout the cell cycle, including M phase. Given that the DSBR and BIR pathways are more active in M phase than in S/G_2_ phase (45, 46), it is reasonable to assume that the relative ratio of T-LOH is higher in cells cultured in furfural-containing medium. Furthermore, the frequency of chromosomal nondisjunction in furfural-treated cells may give rise to chromatid breaks due to an improper pulling force, which must be repaired in M phase, serving as sources of T-LOH.

### Exposure to furfural caused a modest increase in DNA mutations but a significant change in the mutation spectrum.

Several assay systems have previously assessed the mutagenic potential of furfural (8, 47). However, the mechanistic basis of furfural’s mutagenesis has yet to be uncovered. This study demonstrated that exposure to furfural slightly increased the rate of base substitutions in yeast cells. Compared to untreated cells, furfural-treated cells had a significantly higher ratio of CG-to-AT and CG-to-TA mutations in their mutation spectra (Fig. 6A). CG-to-AT substitutions have previously been associated with oxidized guanine and cytosine. 8-oxo-7,8-dihydro-2'-deoxyguanosine (8-oxodG) was found to be the most typical oxidized product of guanine, which tends to pair with adenine (48). If the mismatch is not repaired, a further round of replication results in a G-to-T substitution. The oxidation of cytosine can delaminate the 4-amino group of cytosine, generating uracil (31). The incorporation of A opposite U results in a C-to-T mutation after DNA replication. According to our findings, Ogg1 and Ung1, two glycosidases involved in oxidized base removal (49, 50), are required to reduce point mutations in furfural-treated cells (Fig. 6B). Thus, the higher rates of CG-to-AT and CG-to-TA mutations in furfural-treated cells are likely attributable to the inability of the BER pathway to repair oxidized bases.

### Identification of certain T-LOH events that are associated with furfural.

Using a chromosome IV-specific SNP microarray, we discovered that the breakpoints of furfural-induced T-LOH events were distributed differently on chromosome IV than those occurring spontaneously (Fig. 7B). Most T-LOH events in the red colonies had breakpoints positioned between kb 1050 and the telomere, while the breakpoints of LOH events in white colonies were clustered between the centromere and kb 1050 (Fig. 7B). This finding indicates that some T-LOH events produced daughter cells that were more or less sensitive to furfural and more or less likely to be selected on furfural plates. By creating four mutants with defined breakpoints (kb 950 and kb 1100) of T-LOH using CRISPR/Cas9-mediated DNA breaks on specific sites of chromosome IV, we discovered that the conversion of YJM789-derived *SSD1* to the W303-1A-derived allele resulted in the weaker growth of yeast cells in furfural-containing medium (Fig. 7D). We supported this hypothesis by observing that the introduction of a functional *SSD1* allele in W303-1A significantly improved its furfural tolerance (Fig. 7D). Overall, these results explain why white colonies, in which the W303-1A-derived *SSD1* allele was reconverted to the YJM789-derived allele, are more frequently selected in yeast isolates grown on furfural media.

Hose et al. (38) conducted a study showing that Ssd1p binds to several nucleus-encoded mitochondrial transcripts and can influence the abundance of mitochondrial proteins. The deletion of *SSD1* produced mitochondrial malfunction, which is more noticeable in aneuploid cells (38). Impaired mitochondrial function was identified as a crucial cause of ROS generation (51). The overexpression of *SSD1* was reported to efficiently alleviate ROS accumulation in yeast cells under ethanol stress (52), probably because *SSD1* overexpression enhanced mitochondrial function. Since furfural treatment caused mitochondrial membrane morphologies to transform from tubules to aggregates (28), Ssd1p deficiency would exacerbate furfural’s toxicity to mitochondria, leading to more intense oxidative stress. Overall, our data established a connection between Ssd1p function and furfural tolerance and revealed that any LOH event that affects mitochondrial function and intracellular ROS levels will be favored in yeast cells exposed to furfural.

### Conclusions.

In conclusion, this study demonstrates that nonlethal doses of furfural in media are potent drivers of genomic instability and phenotypic evolution in yeast. In comparison to spontaneous conditions, furfural exposure resulted in a unique pattern of genomic alterations. Our results revealed that aneuploidy and LOH events, which occurred at a rate of 10^−3^ per cell division, served as advantageous evolutionary strategies for diploid yeast cells to achieve adaptive fitness in the presence of furfural. Our findings provide novel insights into the genome evolution of yeast under stressful conditions and offer references for developing robust strains for industrial use.

## MATERIALS AND METHODS

### Strains and medium.

The diploid yeast strains WYUα and JSC25-1 used in this study were created by mating two isogenic haploid strains derived from W303-1A and YJM789 (see Table S1 in the supplemental material). MonchrIII, MonchrV, and MonchrIX were aneuploid mutants in which chromosomes III, V, and IX from the W303-1A-derived genome were deleted, respectively. The deletion in each of these mutants was confirmed by whole-genome sequencing. Haploid strain LSY3877 was a W303-1A-derived strain in which the *CAS9* gene was inserted into the *HIS3* locus of chromosome XV (Table S1). *CAS9* was regulated by the galactose induction promoter *GAL1*. Strain QC97 was generated by crossing LSY3877 with the YJM789-derived strain MD702 (Table S1). The *MAT*α locus in WYUα and JSC25-1 was deleted to prevent meiotic recombination. The deletion of *OGG1* and *UNG1* in the haploid MC42-2d strain (a W303-1A-derived strain with wild-type *CAN1*) resulted in mutants SY112 and SY113, respectively. Strain SY21-1 was a W303-1A-derived mutant in which the *SSD1* allele was replaced with the YJM789-derived allele (Table S1). YPD medium was used, which contained 10 g/L yeast extract, 20 g/L peptone, and 20 g/L dextrose. To prepare plates containing furfural, furfural was added to the medium before the agar solidified, at concentrations ranging from 0 to 0.6 g/L.

### Yeast transformation and gene deletion.

Deletion cassettes for genes *UNG1* and *OGG1* were generated using one-step PCR with the primers listed in Table S2. Plasmids pUG6 and pUG72 were used as the templates to construct deletion cassettes containing the *KanMX6* reporter gene and *URA3*, respectively (53). A lithium acetate (LiAc)/ssDNA/polyethylene glycol (PEG) method was used for yeast transformation (54). The integration of the cassettes was confirmed by PCR (with the primers listed in Table S2) and Sanger sequencing.

### Plasmid construction and *CAS9* induction.

Plasmids gRNA950 and gRNA1100 were used to create CRISPR/Cas9-induced double-strand breaks (DSBs) at kb 950 and kb 1100, respectively, on the right arm of chromosome IV. To construct gRNA950, segments of the template plasmid pAA2 (55) were cloned using two pairs of PCR primers listed in Table S2, resulting in two overlapping PCR products containing the guide RNA coding sequence. These products were assembled into the circular plasmid gRNA950 using a Gibson assembly cloning kit (TransGen Biotech, Beijing, China). gRNA1100 was constructed in a similar way using the primers listed in Table S2.

The transformation of pAA2, gRNA950, and gRNA1100 into strain QC97 resulted in the selection of the leucine-minus transformants QCAA2, QC950, and QC1100. These transformants were incubated in 5 mL 2% galactose medium (containing 10 g/L yeast extract, 20 g/L peptone, and 20 g/L galactose) for 2 h to induce the expression of *CAS9* before being plated onto solid YPD medium.

### Measurement of the frequency of 5-FOA-resistant mutants in WYUα.

To begin, strain WYUα was plated onto YPD plates and incubated at 30°C for 48 h. Cells from a single colony were suspended in 200 μL H_2_O and diluted before being plated onto YPD and furfural-containing YPD plates. The plates were then incubated at 30°C for 3 days (YPD plates) or 5 days (furfural plates) to allow colony formation. Twenty colonies under each condition were suspended in 200 μL double-distilled water (ddH_2_O) and plated onto 20 5-FOA-containing plates. The rate of 5-FOA^R^ mutants was calculated using a method described previously (56). This experiment was performed three times, and the average values are presented.

### Subculture of JSC25-1-derived isolates on furfural plates.

JSC25-1 cells were incubated on solid YPD plates for 48 h at 30°C. Twenty-eight colonies (named “LF” followed by a number) were picked and streaked individually onto YPD plates containing 0.6 g/L furfural to form single colonies. Before the next cycle of furfural exposure, one colony from the furfural-containing plates was purified on a YPD plate for each isolate. This culturing procedure was repeated eight times to accumulate genomic alterations. KQ1 to KQ3 were three isolates that grew for only one cycle on furfural-containing plates.

### White/red sectored colony screening assay.

To determine the effect of furfural on the frequency of sectored colony formation, cells of strain JSC25-1 were incubated on YPD plates at 30°C for 3 days to form colonies. The cells collected from the colonies were diluted and plated onto furfural (0 to 0.6 g/L)-containing plates (~1,000 cells for each plate). Following incubation at 30°C for 5 days, the numbers of sectored colonies were recorded.

### SNP microarray analysis.

A custom whole-genome SNP array (57) and a chromosome IV-specific SNP array (23) were used for LOH analysis of yeast isolates treated with furfural. Genomic DNA extraction from yeast cells and DNA fragmentation (with an average size of about 400 bp) were performed according to a protocol described previously (57). Genomic DNA from the furfural-treated cells was labeled with Cy5-dUTP, and control DNA (from the cells of fully heterozygous strain JSC24-2 [23]) was labeled with Cy3-dUTP. Competitive hybridization of the two DNA samples was performed on the SNP microarrays at 62°C, and the ratio of hybridization of the two differentially labeled samples was analyzed as described previously (57).

### Whole-genome sequencing and analysis.

Whole-genome sequencing of the yeast isolates subcultured on furfural plates was performed on a NovaSeq sequencer using a 2× 150-bp paired-end indexing protocol. The clean reads were mapped onto the yeast reference genome (https://www.yeastgenome.org/) using BWA software (58). SAMtools (59) and VarScan (60) were used to evaluate the copy numbers of W303-1A- and YJM789-derived SNPs. SNVs and indels were detected by using VarScan (60).

### Detection of intracellular ROS.

Yeast cells were precultured in 10 mL of liquid YPD medium for 24 days at 30°C. The cells (~3 × 10^6^) were collected and resuspended in 2 mL of 1× phosphate-buffered saline (PBS) (10 mM phosphate, 138 mM NaCl, and 2.7 mM KCl [pH 7.4]) or 2 mL of 1× PBS containing 0.6 g/L furfural. DCFH-DA was then added to the cell culture to a final concentration of 10 μM (28). After a 1-h incubation at 30°C, the cells were washed with 1× PBS buffer 3 times and resuspended in 1 mL of 1× PBS. A fluorescence spectrophotometer (catalog number RF-6000PC; Shimadzu) was used to measure the fluorescence of yeast cells at a maximum excitation wavelength of 488 nm and a maximum emission wavelength of 525 nm.

### Furfural reduction activity measurement.

Yeast cells were precultured in 15 mL liquid YPD medium overnight. The cells were transferred to 20 mL YPD medium with 0.6 g/L furfural. The yeast cells were collected for protein extraction at the 0-h and 2-h time points by using YeastBuster protein extraction reagent (Merck, Darmstadt, Germany). A bicinchoninic acid (BCA) protein assay kit (Beyotime, Beijing, China) was used to determine the protein concentration. Furfural reduction activity was measured by recording the decreased absorbance at 340 nm using a microplate reader (Tecan M200 Pro NanoQuant). The 600-μL reaction solution contained 0.6 g/L furfural, 0.2 mM NADH, and 100 mM potassium phosphate buffer (pH 7.5). To start the reaction, 20 μL (~1 mg) of the crude extract protein was added to the reaction mixture. One enzyme unit was defined on the basis of 1 μM NADH oxidized per min, and specific activity was described by units per milligram of protein.

### Pulsed-field gel electrophoresis and Southern blot analysis.

Yeast cells were cultured in 7 mL YPD medium overnight. The cells were collected and embedded in plugs (0.8% low-melting-point agarose) containing 1 mg/mL Zymolyase. The plugs were incubated in a 1-mL Tris-EDTA (TE) solution at 37°C for 16 h. Following the addition of proteinase K (6 mg/mL), the incubation mixture was kept at 50°C for 16 h. The plugs were then washed in 10 mL of 1× TE buffer for 3 days before electrophoresis. Yeast chromosomal DNA was separated by pulsed-field gel electrophoresis (PFGE) using a Bio-Rad Chef Mapper system (61) and then transferred to nylon membranes as described previously (62). Hybridization probes were prepared using a digoxigenin (DIG) probe synthesis kit (Roche, Basel, Switzerland) (25). The primers used to synthesize the probes are provided in Table S2. Details of the hybridization and developing conditions were described previously (63).

### Data availability.

All microarray data in this study are available at the GEO database (https://www.ncbi.nlm.nih.gov/geo) under accession numbers GSE132128 (whole-genome arrays) and GSE210078 (chromosome IV-specific arrays). Raw data from Illumina whole-genome sequencing have been deposited in the SRA database (https://www.ncbi.nlm.nih.gov/sra) under BioProject accession number PRJNA416056.
