# Supplementary material for: Nonlethal Furfural Exposure Causes Genomic Alterations and Adaptability Evolution in Saccharomyces cerevisiae
Source: Microbiol Spectr. 2023 Jul 3;11(4):e01216-23. doi: 10.1128/spectrum.01216-23 (PMC10434202; doi:10.1128/spectrum.01216-23)
Supplement: Supplemental file 1 — Supplemental material. Download spectrum.01216-23-s0001.pdf, PDF file, 4.8 MB [file spectrum.01216-23-s0001.pdf]

Supplemental file

Nonlethal furfural exposure causes genomic alterations and phenotypic evolution in *Saccharomyces cerevisiae*

Lei Qi[1,2,3], Ying-Xuan Zhu[2], Ye-Ke Wang[4], Xing-Xing Tang[2], Ke-Jing Li[2], Min He[2], Yang Sui[1,3], Pin-Mei Wang[1,2], Dao-Qiong Zheng[1,2,*], Ke Zhang[5,*]

[1] Donghai Laboratory, Zhoushan, 316021, China

[2] Ocean College, Zhejiang University, Zhoushan, 316021, China

[3] Department of Molecular Genetics and Microbiology, Duke University, Durham, NC, 27705, USA

[4] Life Sciences Institute, Zhejiang University, Hangzhou, 310058, China

[5] College of Life Science, Zhejiang University, Hangzhou, 310058, China

*Corresponding author: zhengdaoqiong@zju.edu.cn (DQ-Z) or zhangke726@zju.edu.cn (KZ)

Table S1. Yeast strains used in this study.

Strain	Background	Construction	Genotype
JSC25-1	W303-1A/YJM789	[1]	*MAT***a***/MATα::HYG ade2-1/ade2-1 can1-100Δ::natMX4/ CAN1Δ::natMX4 ura3-1/ura3 trp1-1/TRP1 his3-11,15/HIS3 leu2-3,112/LEU2 RAD5/RAD5 IV1510386::kanMX6-can1-100/IVI1510386::SUP4-o GAL2/gal2*
JSC24-2	W303-1A/YJM789	[1]	*MAT***a***/MATα::natMX4 ura3/ura3-1 ade2-1/ade2-1 trp1-1/TRP1 his3-11,15/HIS3 leu2-3,112/LEU2 RAD5/RAD5 IV1510386:kanMX6-can1-100/IVI1510386::SUP4-o GAL2/gal2*
W1588-4c	W303-1A	[1]	*MAT***a** *leu2-3,112 his3-11,15 ura3-1 ade2-1 trp101 can1-100 RAD5*
JSC20-1	YJM789	[1]	*MATα ade2-1 ura3 gal2 ho::hisG IV1510386::SUP4-o*
JSC21-1	YJM789	[1]	*MATα leu2::hphMX ura3 can1::natMX gal2 ade2-1 ho::hisG IV1510886::SUP4-o*
W303U	W303-1A	Insertion of the *URA3* gene in the right arm of chr IV of W1588-4c	*MAT***a** *leu2-3,112 his3-11,15 ura3-1 ade2-1 trp101 can1-100 RAD5 IV1168918::URA3*
WYU	W303-1A/YJM789	Crossing W303U and JSC20-1	*MAT***a***/MATα LEU2/leu2-3,112 HIS3/his3-11,15 ura3/ura3-1 ade2-1/ade2-1 TRP1/trp1-1 CAN1/can1-100 IV1168918/IV1168918::URA3*
WYUα	W303-1A/YJM789	Deletion of *MATα* in WYU	*MAT***a***/MATα::hyg LEU2/leu2-3,112 HIS3/his3-11,15 ura3/ura3-1 ade2-1/ade2-1 TRP1/trp1-1 CAN1/can1-100 IV1168918/IV1168918::URA3*
MC42-2d	W303-1A	[2]	*MAT***a** *leu2-3,112 his3-11,15 ura3-1 ade2-1 trp101 RAD5*
SY112	W303-1A	Deletion of *OGG1* in MC42-2d	*MAT***a** *leu2-3,112 his3-11,15 ura3-1 ade2-1 trp101 ogg1 RAD5*
SY113	W303-1A	Deletion of *UNG1* in MC42-2d	*MAT***a** *leu2-3,112 his3-11,15 ura3-1 ade2-1 trp101 ung1 RAD5*
LSY3877	W303-1A		*MAT***a** *RAD5 leu2-3,112 trp1-1 ura3 can1 his3::CAS9-FLAG-HIS5 ade2-1*
MD702-3	YJM789	Deletion of *LEU2* in JSC21-1	*MATα leu2::hphMX ura3 can1::natMX gal2 ade2-1 ho::hisG IV1510886::SUP4-o*
MD703-3	W303-1A/YJM789	Crossing LSY3877 and MD702-3	*MAT***a***/MATα leu2-3,112/leu2::hphMX trp1-1/TRP1 ura3/ura3 can1/can1::natMX his3::CAS9-FLAGHIS5/HIS3 ade2-1/ade2-1 GAL2/gal2 IV1510886/IV1510886::SUP4-o*
QC97	W303-1A/YJM789	Deletion of *MATα* in MD703-3	*MAT***a***/MATα::HYG leu2-3,112/leu2::hphMX trp1-1/TRP1 ura3/ura3 can1/can1::natMX his3::CAS9-FLAGHIS5/HIS3 ade2-1/ade2-1 GAL2/gal2 IV1510886/IV1510886::SUP4-o*
YX-850	W303-1A/YJM789	Transformation of plasmid gRNA850 in QC97	*MAT***a***/MATα::HYG leu2-3,112/leu2::hphMX trp1-1/TRP1 ura3/ura3 can1/can1::natMX his3::CAS9-FLAGHIS5/HIS3 ade2-1/ade2-1 GAL2/gal2 IV1510886/IV1510886::SUP4-o*

YX-1100	W303-1A/YJM789	Transformation of plasmid gRNA1100 in QC97	*MAT***a***/MATα::HYG leu2-3,112/leu2::hphMX trp1-1/TRP1 ura3/ura3 can1/can1::natMX his3::CAS9-FLAGHIS5/HIS3 ade2-1/ade2-1 GAL2/gal2 IV1510886/IV1510886::SUP4-o*

Table S2. Primers used in strain constructions or strain analyses.

Primer	Sequence	Purpose
InURA3S	CAGCTGTCATTTCGCTTTTGTTTTCTCCCAC<br>TCATCGCAAGTTGTACTGAGAGTGCACC	Amplification of *URA3* gene that was inserted at coordinate 1168918 on IV
InURA3A	TAGTACCGCTTTTTTTCGTTGTTTTTTTGTTG<br>TTGAGGAGGCATCTGTGCGGTATTTCA	
vURA3S	GCTATTCGTGAATCTCAAGG	Verification of *URA3* insertion on IV
vURA3A	TTGAGCAATAAAGCCGATA	
vChrIVMonS	AACCTTTAACATTCAGGGAG	Detect the heterozygosity of the left arm of chr IV
vChrIVMonA	ATGACTGCTTGGTAGTTGAG	
dMATS	AATCGTCCTGTCCCATTACG	Replacement of *MATα* with *HYG*
dMATA	TTGGAAACACCAAGGGAGAG	
MATR	AGTCACATCAAGATCGTTTATGG	Primers used for PCR analysis of mating type
MATαF	GCACGGAATATGGGACTACTTCG	
MATaF	ACTCCACTTCAAGTAAGAGTTTG	
vchrII200kS	AACACCTACACGCCTCCT	Amplification of probe targeted at 200 kb of chr II for southern blot
vchrII200kA	GGAAGTAGCGAAGGATGG	
vchrIII250kS	TGTCACGCCATACAGAGGG	Amplification of probe targeted at 250 kb of chr III for southern blot
vchrIII250kA	TCATACCGCCTTGGCTACTT	
vchrXIII100kS	CGTGATGTTGAGCGAGATAG	Amplification of probe targeted at 100 kb of chr XIII for southern blot
vchrXIII100k<br>A	GAGGGCTTGATAATGTGGT	
vchrIX160kS	GTGCGAAGACCGTTCAAGTG	Amplification of probe targeted at 160 kb of chr IX for southern blot
vchrIX160kA	GAACAGCGTGTCCCAGGTAA	
dOGG1S	AATTGCGATTTTATTTATCAACCAGATGTCT<br>TATAAATTCGGCAGGTCGACAACCCTTA	Amplification of *OGG1* knockout cassette using plasmid pUG6 as the template
dOGG1A	TACCACTAGTCCCTCCGATTTCTTTAGAGA<br>ATAGGACACCTTGGATCTGATATCACCTA	
vOGG1S	ATGCGTCAATCGTATGTG	Verification of *OGG1* deletion
vOGG1A	TGTGAGACCACCTATTGAAG	
dUNG1S	ATTATCAGAAGCTGTACACAAGCCGTTCAC<br>ATACATGTGGTGCATCAGAGCAGATTGTA	Amplification of *UNG1* knockout cassette using plasmid pSH47 as the template
dUNG1A	AAATCGAGACCTGCATATGCAATAGTAATA<br>TTCAAGGGTCCTTTAGTTTTGCTGGCCGC	
vUNG1S	TTAGATGACAAGGGAGACGC	Verification of *UNG1* deletion
vUNG1A	GTTGGTTTCGTTCCAGTCAG	
SNR52-S1	AGGTTTCCCGACTGGAAAGGTATGACCATG<br>ATTACGCCAAGC	Amplification of a segment from the plasmid pAA2 to construct the plasmid gRNA850
g950kb(2) D-<br>A	CGCAAGAAATCGACTCTCGG<br>GATCATTTATCTTTCACTGCGG	
g950kb(2) D-S	CCGAGAGTCGATTTCTTGCG<br>GTTTTAGAGCTAGAAATAGCAAGTT	Amplification of a segment from the plasmid pAA2 to construct the plasmid
sca-2u-A	TTTCCTTTGATATTGGATCGACTAGTGGAG	

	GGCGTGAACGTAAGCGTGACA	gRNA850
g110kb(2)D-A	TGCCCTTGCACGATTACGAT GATCATTTATCTTTCACTGCGG	Amplification of a segment from the plasmid pAA2 to construct the plasmid gRNA1100, paired with SNR52-S1
g110kb(2)D-S	ATCGTAATCGTGCAAGGGCA GTTTTAGAGCTAGAAATAGCAAGTT	Amplification of a segment from the plasmid pAA2 to construct the plasmid gRNA1100, paired with sca-2u-A

Table S3. Point mutations detected in 21 sequenced JSC25-1-derived isolates

NO.	Isolate	Chromosome	Location	Reference	Mutation
1	LF14	chrII	294371	A	G
2	LF3	chrII	638763	C	A
3	LF3	chrIII	54814	G	T
4	LF18	chrIII	305770	G	A
5	LF14	chrIII	316156	C	A
6	LF14	chrIV	29124	T	C
7	LF8	chrIV	210303	C	T
8	LF18	chrIV	803878	T	C
9	LF14	chrIV	1160655	G	A
10	LF14	chrIV	1504520	T	A
11	LF14	chrV	498534	G	T
12	LF18	chrVI	13252	T	C
13	LF14	chrVII	77677	T	C
14	LF20	chrVII	842368	G	A
15	LF20	chrVIII	144769	G	T
16	LF14	chrVIII	533291	C	T
17	LF20	chrVIII	535379	G	T
18	LF18	chrXI	664644	T	C
19	LF18	chrXII	815478	C	T
20	LF8	chrXIII	30229	T	A
21	LF8	chrXIII	159047	G	A
22	LF10	chrXV	125492	C	A
23	LF8	chrXVI	173117	G	C
24	LF20	chrXVI	700576	C	T
25	LF10-28	chrXVI	316942	G	T
26	LF10-21	chrXII	185738	G	T
27	LF10-21	chrXV	237006	G	A
28	LF10-22	chrVIII	309292	G	A
29	LF10-22	chrXV	237006	G	A
30	LF10-22	chrXV	427911	G	T
31	LF10-22	chrXVI	767313	C	T
32	LF10-23	chrIX	414521	C	T
33	LF10-23	chrX	693237	C	A
34	LF10-23	chrXV	577503	C	T
35	LF10-24	chrI	98745	G	A
36	LF10-24	chrIII	184947	G	A

37	LF10-24	chrIX	126556	T	G
38	LF10-24	chrV	329058	A	G
39	LF10-25	chrII	272146	G	T
40	LF10-25	chrVI	248730	C	A
41	LF10-25	chrVIII	236761	A	T
42	LF10-25	chrXIV	298408	A	C
43	LF10-25	chrXV	694707	G	T
44	LF10-25	chrXV	882315	A	G
45	LF10-26	chrIV	1351113	T	G
46	LF10-26	chrV	208578	T	G
47	LF10-26	chrXII	84621	G	C
48	LF10-26	chrXII	84622	A	G
49	LF10-26	chrXVI	431944	C	G
50	LF10-26	chrXVI	786041	C	T
51	LF10-27	chrI	12791	C	T
52	LF10-27	chrI	12793	G	A
53	LF10-27	chrXIV	138687	G	C
54	LF18	chrVII	807474	G	GGATGAA
55	LF8	chrVII	1082863	GA	G
56	LF16	chrXVI	76565	C	CA

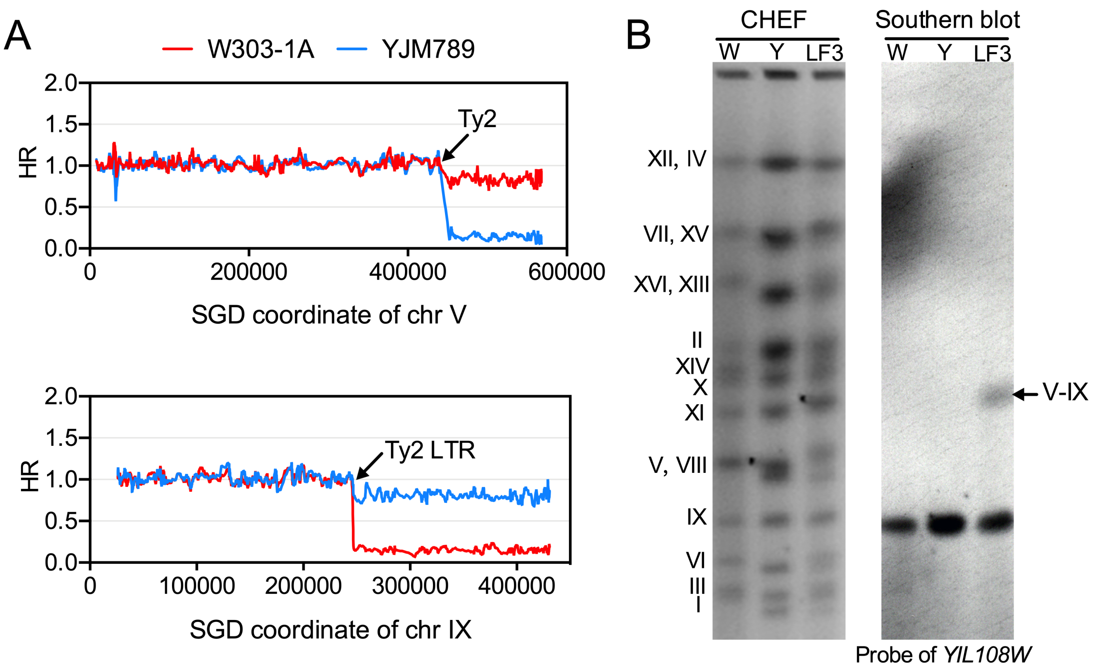

Fig. S1. Translocation event detected in the isolate LF3. (A). The terminal deletions on chromosomes V and IX were disclosed using whole-genome SNP microarray. (B). Plus-field gel electrophoresis and Southern blot analysis showed the segments of chromosome V (0-443 kb) and IX (0-246 kb) were fused to generate a new chromosome (V-IX). The probe of Southern blot analysis was designed to detect *YIL108W* gene on chr IX.

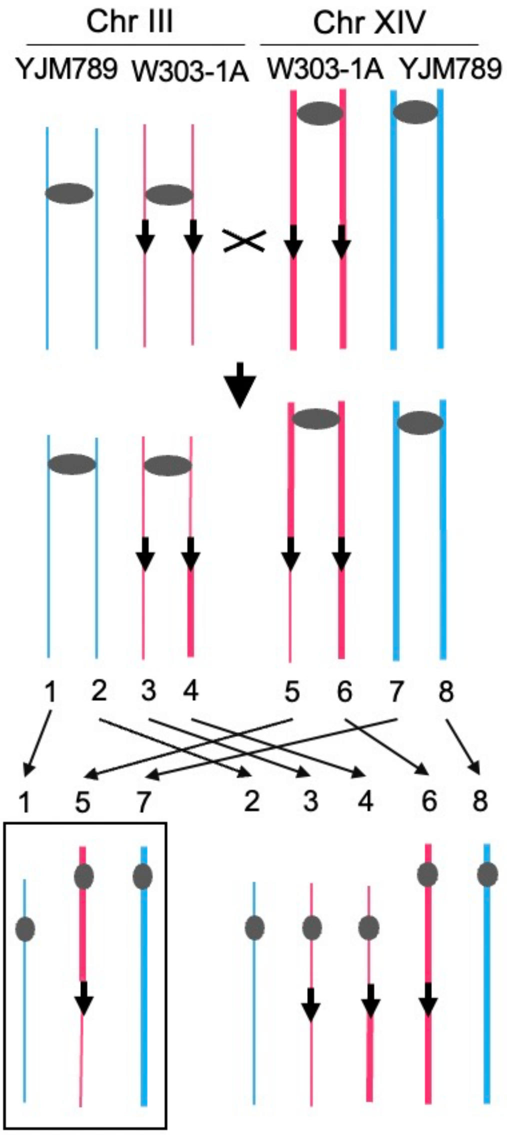

Fig. S2. A genetic model of the translocation event in LF3. W303-1A- and YJM789-derived homologs are shown as red and blue lines. Chromosomes III and XIV are depicted by thin and thick lines respectively. Centromere and Ty1 elements are represented by gray oval and black arrow. A crossover between W303-1A-derived chromosomes III and XIV following unequal chromatid segregation will give rise the "paired" deletion in the daughter cell indicated by the boxed pattern.

References

1. St Charles J, Petes TD. 2013. High-resolution mapping of spontaneous mitotic recombination hotspots on the 1.1 Mb arm of yeast chromosome IV. PLoS Genet 9:e1003434.
2. Zheng DQ, Wang YT, Zhu YX, Sheng H, Li KJ, Sui Y, Zhang K. 2022. Uncovering bleomycin-induced genomic alterations and underlying mechanisms in the yeast *Saccharomyces cerevisiae.* Appl Environ Microbiol 88:e0170321.
